# Single Cell Oil Production by Oleaginous Yeasts Grown in Synthetic and Waste-Derived Volatile Fatty Acids

**DOI:** 10.3390/microorganisms8111809

**Published:** 2020-11-17

**Authors:** Sara Bettencourt, Catarina Miranda, Tatiana A. Pozdniakova, Paula Sampaio, Ricardo Franco-Duarte, Célia Pais

**Affiliations:** 1 CBMA—Centre of Molecular and Environmental Biology, Department of Biology, University of Minho, Campus de Gualtar, 4710-057 Braga, Portugal; bettencourtsmp@gmail.com (S.B.); catarinacmiranda@hotmail.com (C.M.); psampaio@bio.uminho.pt (P.S.); ricardofilipeduarte@bio.uminho.pt (R.F.-D.); cpais@bio.uminho.pt (C.P.); 2 IB-S—Institute of Science and Innovation for Bio-Sustainability, University of Minho, Campus de Gualtar, 4710-057 Braga, Portugal

**Keywords:** lipids, single cell oils, volatile fatty acids, dark fermentation effluent, oleaginous yeasts

## Abstract

Four yeast isolates from the species—*Apiotrichum brassicae*, *Candida tropicalis*, *Metschnikowia pulcherrima*, and *Pichia kudriavzevii*—previously selected by their oleaginous character and growth flexibility in different carbon sources, were tested for their capacity to convert volatile fatty acids into lipids, in the form of single cell oils. Growth, lipid yields, volatile fatty acids consumption, and long-chain fatty acid profiles were evaluated in media supplemented with seven different volatile fatty acids (acetic, butyric, propionic, isobutyric, valeric, isovaleric, and caproic), and also in a dark fermentation effluent filtrate. Yeasts *A. brassicae* and *P. kudriavzevii* attained lipid productivities of more than 40% (*w*/*w*), mainly composed of oleic (>40%), palmitic (20%), and stearic (20%) acids, both in synthetic media and in the waste-derived effluent filtrate. These isolates may be potential candidates for single cell oil production in larger scale applications by using alternative carbon sources, combining economic and environmental benefits.

## 1. Introduction

The global warming crisis has increased the demand for renewable materials production, with the capacity to replace petroleum-based compounds, boosting, in this way, the circular economy strategy to reuse and recycle. The main focus in previous years was the sustainable processing of biomass into a spectrum of biobased products with applications in food and bioenergy industries [1], mainly oleochemicals (chemicals originating from plant oils and animal fats). Oleochemical applications are diverse, ranging from surfactants, lubricants, plastics, biofuels to cosmetics, amidst others [2,3].

The necessity to replace petrochemicals with eco-friendly compounds, such as basic oleochemical substances, has increased researchers’ attention on the production of microbial oils—single cell oils (SCOs)—since they are identical in type and composition to those oils and fats from plants or animals [4]. However, the current industrial production of SCOs is not yet economically competitive due to the low productivity of many oleaginous microorganisms, together with the high costs associated with the fermentative process. Recent research has tried to overcome these problems, both by the search of new efficient microbial producers with increased lipid production yield and/or by the use of alternative low-cost renewable substrates, both increasing the commercial potential of SCOs bioproduction.

Among the group of oleaginous microorganisms, yeasts are oil producers par excellence. This attribution owes to their special characteristics, such as high lipid content, short duplication times, ability to grow on an extensive range of substrates and carbon sources, light independence, and low pH tolerance [2,5]. Considering the entire yeast population, oleaginous yeasts represent a minority part, with just 5% of them being capable of accumulating more than 20% of lipids as lipid bodies (the minimum amount to be considered as oleaginous) [6]. The most described oily yeast genera comprise *Yarrowia*, *Candida*, *Rhodotorula*, *Rhodosporidium*, *Cryptococcus*, *Trichosporon*, and *Lipomyces* [7]. In 2014, 70 oleaginous yeast species were described as oil-bearing microorganisms [5]. In our previous work [8], we developed a screening method to rapidly detect oleaginous species, which allowed the species *Millerozyma farinosa*, *Trigonopsis cantarellii*, and *Geotrichum candidum*, for the first time, to be defined as oleaginous, and to confirm the oleaginous character of many others. Notwithstanding, it is worth mentioning that lipid content and profile may not be species-dependent, varying amongst strains of the same species, as reported in recent works [8,9].

A plethora of substrates are becoming increasingly exploited as cheap and renewable substrates to reduce costs associated with SCOs production [10]. Molasses, wastes from industries, and wastewater are some of the fermentation substrates used for microbial oils production [11,12]. The degradation of organic wastes biomass can originate volatile fatty acids (VFAs), which can be exploited as carbon sources for lipid production [13]. This conversion can occur via anaerobic digestion. Waste rich in moisture and organic matter, such as municipal solid waste, is the perfect substrate for anaerobic digestion [14]. Generation of this type of waste is registering an alarming increase. In the 2012 global review of solid waste management, 624,700 tons of municipal solid wastes were reported to be generated daily in the United States, 520,548 in China, and 13,616 in Portugal [15]. Municipal solid waste is normally disposed in sanitary landfills, its reduction and reuse being mandatory [14]. Additionally, the poor management of waste influences health, the environment, and economy, being imperative to act to improve it. VFAs resultant from anaerobic digestion processes consist of mainly acetic (HAc), propionic (HPr), and butyric (HBut) acids and in lower concentration, isobutyric (isoHBut), valeric (HVal), isovaleric (isoHVal), and caproic (HCap) acids. The increasing interest in these short-chain fatty acids is related to their applicability in cost-effective microbial lipid production and, simultaneously, in waste utilization and bioremediation [16]. Additionally, VFAs are characterized by their short metabolic pathways. It is already known that different types of VFAs and variable concentrations have different inhibitory effects on cell growth and lipid accumulation. Some authors have reported the production of SCOs by yeasts using acetic, propionic, and butyric acids as sole carbon sources [16,17,18], and a few studies also tested the mixture of HAc:HPr:HBut [16,17]. However, to the extent of our knowledge, no study has been published yet testing the mixture of HAc:HPr:HBut:isoHBut:HVal:isoHVal:HCap, which is of great importance to assess, these being the main acids present in digestates resultant from anaerobic processes. Some authors have already tested real dark effluent filtrates as a substrate for microbial production of SCOs; however, lower lipid content was obtained when compared with synthetic VFAs [16,19].

The oleaginous yeasts’ long-chain fatty acids (LCFAs) profile depends on several factors, especially medium composition and culture conditions, also varying with the yeast species and strains [20]. Nevertheless, oleic (C18:1) and linoleic (C18:2) acids, together with palmitic (C16:0) or palmitoleic (C16:1) acids, are the common fatty acids produced [19,21]. Considering the LCFAs profile, applications of yeast oils can be diverse, namely in food, pharmaceutical, and biofuel industries [22]. The interest in yeasts, as sources of edible lipids, has centered principally on the ability of these microorganisms to synthesize lipids rarely found in the plant or animal kingdom. This is the case of lipids found in cocoa-butter and to a lesser extent that of other exotic fats like shea butter, which can be synthesized in large amounts by oleaginous yeasts when grown in appropriate substrates [23].

In terms of large-scale applications, only the ones related to the production of polyunsaturated fatty acids in high concentrations have been exploited in recent years [24,25]. However, other types of lipids, with a more common composition, have aroused interest and should start to be produced on a larger scale in the near future [26,27].

The main objective of the present work was to evaluate the efficiency of four yeast species—*Apiotrichum brassicae*, *Candida tropicalis*, *Metschnikowia pulcherrima*, and *Pichia kudriavzevii*—previously defined as oleaginous, to metabolize VFAs, both in a synthetic mixture or using a real dark fermentation effluent filtrate. Yeast performance was compared in terms of biomass production, lipid yields, VFAs consumption, and LCFAs profiles, concluding about their potential to be used in a larger scale.

## 2. Materials and Methods

### 2.1. Yeast Isolates

Four yeast isolates were used in this study, belonging to the following species (isolate numbers indicated after species name): *Apiotrichum brassicae* V134 (GenBank accession number MN913458), *Candida tropicalis* V139 (GenBank accession number MN913460), *Metschnikowia pulcherrima* V213 (GenBank accession number MN913467), and *Pichia kudriavzevii* V194 (GenBank accession number MN913463).

The yeasts were maintained for long term conservation at −80 °C in glycerol 30% (*v*/*v*), and were grown out of cryopreserved stocks using YPD medium (0.5% Yeast extract (*w*/*v*; Panreac AppliChem, Darmstadt, Germany), 1% Peptone (*w*/*v*; BD biosciences, San Jose, CA, USA), 2% Dextrose (*w*/*v*; Scharlau, Barcelona, Spain)), supplemented with agar 2% (*w*/*v*; LabChem, Zelienople, PA, USA), prior to each experiment.

### 2.2. Culture Media

To evaluate the effect of different VFAs in yeast growth and lipid production, experiments were conducted in YP medium (0.5% Yeast extract (*w*/*v*), 1% Peptone (*w*/*v*)) supplemented with different synthetic VFAs as carbon sources. The composition of the culture media used throughout the work is detailed in Table 1. Five different synthetic VFAs were considered (as sole carbon sources and/or as a mixture) and concentrations were chosen based on the composition of a real dark fermentation effluent, whose composition is also presented in Table 1, and that was used later in the work. Synthetic VFAs used as carbon sources were glacial acetic acid (99.7% Panreac AppliChem, Darmstadt, Germany), propionic acid (>99.0% Merck, Darmstadt, Germany), butyric acid (>99.0% Merck, Darmstadt, Germany), isobutyric acid (>98.0% Merck, Darmstadt, Germany), and valeric acid (>99.0% Merck, Darmstadt, Germany). Each VFAs solution was prepared separately and the pH was adjusted to 7.0 with sodium hydroxide (1 and 12 M). After, in aseptic conditions, the VFAs solutions were sterilized by vacuum filtration through mixed cellulose ester filters with 0.22 μm of pore diameter (47 mm, Millipore, Darmstadt, Germany) and added to the previous autoclaved YP solutions.

After demonstrating the efficient utilization of the synthetic VFAs by the four yeast species, fermentation with a real dark fermentation effluent was tested. The effluent liquid fraction was derived from vegetable garden food waste, provided by Organic Waste Systems NV (OWS, Gent, Belgium) and processed by Tecnalia Research & Innovation (Vitoria-Gasteiz, Spain). The effluent was sterilized using vacuum filtration with a 0.22 μm mixed cellulose ester sterile filter (47 mm, Millipore), under aseptic conditions and hereafter designated by effluent filtrate. The effluent filtrate characterization is present in Table 1. 

### 2.3. Batch Culture Conditions

Yeasts were pre-cultured in YPD medium and incubated for 24 h (30 °C, 170 rpm). After this period, cultures were harvested by centrifugation, washed once using phosphate buffered saline 1× (PBS), and inoculated into different fermentation media (Table 1) with an initial optical density at 640 nm (OD_640_) of 0.6. Batch cultures were performed in 100 mL Erlenmeyer flasks with 40 mL culture medium for 120 h, at 30 °C, and 170 rpm. The batch cultures were performed in triplicate in order to assure the reproducibility of the results.

### 2.4. Analytical Methods

#### 2.4.1. Biomass and Lipids Quantification

Yeast biomass was measured as OD_640_ and by dry cell weight (DCW). Intracellular lipid accumulation was estimated by a fluorescence assay using the lipophilic dye Nile red (>98.0% Sigma Aldrich, Steinheim, Germany) and quantified as percentage of lipids by DCW (% *w*/*w*), according to Miranda et al. [8].

#### 2.4.2. Volatile Fatty Acids Quantification

High-performance liquid chromatography with refractive index (HPLC-RI) was used to quantify VFAs. Prior to analysis, protein contaminants were removed by mixing 1 mL of sample with 100 µL of 100% (*w*/*v*) trichloroacetic acid (99.5% Panreac AppliChem, Darmstadt, Germany) and stored at 4 °C for 2 h. The samples were then centrifuged at 4 °C, 13,000 rpm for 15 min and the supernatant was filtered through 0.22 µm filters into HPLC vials. Samples were analyzed with a HyperREZ™ XP Carbohydrate H+ 8 µm (Thermo Electron Corporation, Waltham, MA, USA) column on a LaChrom Elite^®^ (VWR Hitachi, Tokyo, Japan) chromatography system with a LaChrom Elite^®^ L-2490 RI detector (VWR Hitachi, Tokyo, Japan). The mobile phase was a sulfuric acid solution (2.5 mM) prepared in ultra-pure water with a flow rate of 0.5 mL min^−1^ for 90 min. The operation temperatures were 40 °C for the column and 45 °C for the detector. The percentage of VFAs degradation was determined by comparing the peak areas at t = 120 h with the ones obtained at t = 0 h. Data were expressed as the mean of three replicates ± standard deviations.

#### 2.4.3. Total Organic Carbon and Nitrogen Quantification

Total dissolved nitrogen (TN) was measured by 720 °C catalytic thermal decomposition/chemiluminescence methods and total dissolved organic carbon (TOC) was determined by the combustion catalytic oxidation method, at 680 °C, in a SHIMADZU TOC-L/TNM-L analyzer.

#### 2.4.4. Lipid Profile Analysis (Fatty Acid Methyl Esters, FAME)

Extracted lipids were subjected to an acid-catalyzed transesterification process prior to analysis by gas chromatography with a flame ionization detector (GC-FID). Firstly, lipids were dissolved in 4 mL of n-hexane (99.0% Panreac AppliChem, Darmstadt, Germany). For the transesterification step, 1 mL of the diluted sample, 100 μL of heptadecanoic acid (internal standard, 98.0% Sigma Aldrich, Steinheim, Germany), and 1 mL of the mixture 10% MeOH:H_2_SO_4_ (10:1 (*v*/*v*)) were added to glass capped test tubes with a screw cap and heated at 100 °C for 2 h. The organic phase was collected and after residual water removal with Na_2_SO_4_, fatty acid methyl esters were analyzed by GC-FID (CP-3800 Varian, Agilent, Santa Clara, CA, USA) using a Teknokroma^®^ TR WAX 30 m × 0.25 mm × 0.25 µm column. GC-FID operational conditions were as follows: carrier gas flow (He) 1 mL min^−1^; injector—250 °C; detector—280 °C; oven—40 °C with a ramp of 30 °C min^−1^ up to 150 °C followed by a ramp of 3 °C min^−1^ up to 250 °C. The volume of the injected sample was 1 µL and the split (1:10) injection technique was applied. For peak identification, a mixture containing saturated, monounsaturated, and polyunsaturated FAME ranging from C4:0 to C24:1 was used (Supelco^TM^ 37 Component FAME Mix, Sigma Aldrich, Steinheim, Germany). Results were expressed as percentages and as means of triplicates ± standard deviations.

#### 2.4.5. Statistical Analysis

One-way parametric ANOVA was used to evaluate yeast growth and lipid production, using the software MINITAB (version 19.2020.1, State College, PA, USA). Differences were considered as statistically significant at a *p*-value lower than 0.01 (1%).

## 3. Results and Discussion

In order to be candidates for large scale production of SCOs, oleaginous yeasts, in addition to having a considerable lipid productivity, also need to be able to grow to high cell densities, with increased biomass accumulation capacities. Moreover, oleaginous yeasts able to grow on a broad array of carbon sources increase their economic interest. For the present work, four yeast species were chosen, based on lipid production and biomass results, previously obtained in glucose and acetic acid media [8]: *Apiotrichum brassicae* V134, *Candida tropicalis* V139, *Metschnikowia pulcherrima* V213, and *Pichia kudriavzevii* V194. *A. brassicae* is a synonym of the already described oleaginous yeast *Trichosporon brassicae* [28]; however, exploitation of its oleaginous character is still scarce, despite the oil-bearing character of the *Apiotrichum* genus described in previous studies [29,30]. Regarding the *Candida* genus, several species were reported as oil producers, namely *C. curvata* [31], *C. bombicola* [32], and *C. viswanathii* [10], amidst others. The oil accumulation properties of *C. tropicalis* had also been explored by some authors [33,34]. Within the genus *Pichia*, some species have been described as good oil producers [35,36]. However, the oleaginous character of *P. kudriavzevii* is not frequently exploited in the literature [37,38]. *M. pulcherrima* was first described as an oleaginous microorganism over more than a hundred years ago [39], but its oleaginous potential is still scarcely explored.

Considering these aspects, different experiments were conducted to determine the best conditions for SCOs accumulation in the four selected isolates.

### 3.1. Time Course Growth and SCOs Production in Acetic Acid

Growth profile, lipid production, VFAs consumption, and LCFAs composition were evaluated in a continuous experiment, carried out in YP media with acetic acid (15 g L^−1^). Experiments were performed in a time course, along 168 h, in order to confirm the best conditions for improved production of SCOs by the four yeasts. Acetic acid was the carbon source used since it is commonly the main VFA present in mixtures obtained through anaerobic digestion of organic wastes. Several authors have already assessed this characterization [10,12], though not with the same yeast species, media, and culture conditions as the ones used in this work.

Two pH values were tested: pH 6.9 ± 0.1 as the one closer to the pH of the effluent filtrate, and 5.5 ± 0.1 as an acidic pH that can prevent contaminations and used previously in lipid production screenings [40]. However, at pH 5.5, only two yeasts were able to grow (results in Figure S1, Tables S1 and S2)—*C. tropicalis* V139 and *M. pulcherrima* V213; lipid production was lower than what was expected (≤20% *w*/*w*) and LCFAs profiles did not produce relevant differences. In this way, only the pH value of 6.9 was considered throughout the remainder of the work as being the most suitable one to fulfill the research objectives.

**Figure 1:**
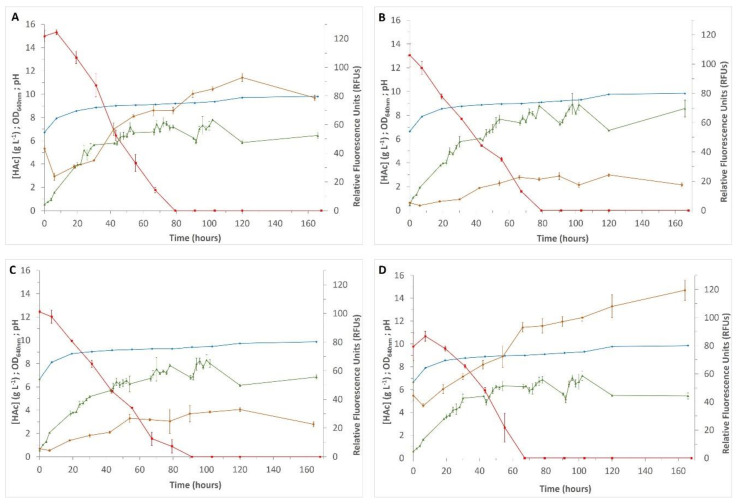
Time-course growth in YP:HAc 15 g L^−1^ medium, during 168 h, using the four tested yeast species: (**A**) *Apiotrichum brassicae* V134; (**B**) *Candida tropicalis* V139; (**C**) *Metschnikowia pulcherrima* V213; (**D**) *Pichia kudriavzevii* V194. Colors indicate measurements (mean ± standard deviations) of the following characteristics: orange—relative fluorescent units (RFUs); green—optical density (OD_640 nm); blue—pH; red—acetic acid concentration (g L^−1^).

Results obtained for the four selected yeasts, when growth was performed in YP:HAc (15 g L^−1^) at pH 6.9 for 168 h, are shown in Figure 1. As is visible, all strains were able to grow and produce lipids. At that pH, the lag phase was practically inexistent, a notorious difference when compared to the results obtained at a lower pH. The absence of a pronounced lag phase suggested that cells quickly adapted to the new medium, a fact that was also corroborated by a short decline in the RFUs measured. The initial RFUs (t = 0 h) translate the lipids that yeasts accumulated during the pre-inoculum period in glucose-rich medium and the decrease observed (t = 7 h) was triggered by an adaptation to the new environment, where cells consumed the lipids previously synthesized before starting depletion of the new carbon source. The time course profile also showed that the beginning of acetic acid degradation was accompanied by an increase in the medium pH. The total exhaustion of acetic acid from the media started after 80 h of growth for *A. brassicae* V134 and *P. kudriavzevii* V194 and for strains *C. tropicalis* V139 and *M. pulcherrima* V213, acetic acid was only completely consumed after around 90 h.

Concerning lipid production, a dissimilar production of SCOs could be observed along growth time. When using *A. brassicae* V134 and *P. kudriavzevii* V194, the lipid content continued to increase exponentially during the stationary phase, while a slower lipid accumulation was observed for *C. tropicalis* V139 and *M. pulcherrima* V213. However, the highest production point was achieved at 120 h for almost all the strains (with the exception of *P. kudriavzevii* V194, in which a slight increase was detected at 168 h), confirming that this time point is the one ensuring the highest lipid accumulation. At 120 h of growth, SCOs production reached noteworthy values, especially for *A. brassicae* V134 and *P. kudriavzevii* V194 (approximately 93 and 108 RFUs, respectively), obtaining statistically significant differences (*p* < 0.01) in comparison with the other two strains. In this way, this time point (120 h) is recommended to obtain maximum SCOs production by the selected yeasts and it was used throughout the rest of the work. Similar results were observed by Dourou et al. [41], concluding about the kinetics of lipid accumulation in *Yarrowia lipolytica*, but in glucose containing medium. This reduction in lipid accumulation could be due to the fact that, at 120 h, yeasts reached the lipid turnover phase, the point where the *β*-oxidation pathway is triggered to remobilize the carbon that has been stored [42]. When cells entered the stationary phase (approximately 54 h for all strains), acetic acid was still available in the medium. Therefore, it was possible to conclude that, globally, consumed carbon was channeled toward intracellular storage compounds, instead of being used for cellular growth and biomass production, an observation that is in accordance with the increase in RFUs.

In fact, two central pathways are responsible for the lipid accumulation inside the yeast cell designated as ex novo and de novo lipid synthesis. The activation of the ex novo synthesis pathway requires hydrophobic substrates as carbon sources in the culture medium, and is completely independent of nitrogen deprivation conditions. During this growth-associated process, fatty compounds in general (e.g., oils, free fatty acids, and triacylglycerols) are incorporated from the culture medium to be subsequently internalized in the cell. However, this is a multi-step procedure and, therefore, it comprises the degradation of hydrophobic substrates outside the cell via secretion of lipases, transport of free fatty acids inside the cell by active transport, and the subsequent modification of the fatty acids within the cell. Posteriorly, these molecules can be used either for cell propagation or for the transformation of new fatty acids that were not present in the medium before. The observed lipid degradation is reported here for the first time in these yeast species. Nonetheless, it seems to be a common feature for many oleaginous types of yeasts and filamentous fungi such as *Y. lipolytica* [43,44], *Cryptococcus curvatus* [45], and *Trichoderma viridae* [46].

Regarding biomass production, lipid content, lipid output, and lipid yield, results are summarized in Table 2 (“YP:HAc 15 g L^−1^”) for the four yeast species. Isolates *C. tropicalis* V139, *P. kudriavzevii* V194, and *M. pulcherrima* V213 presented high DCW values, between 3.51 and 3.86 g L^−1^ (not significantly different), while *A. brassicae* V134 biomass was about 30% lower (statistically significant at *p* < 0.01). The highest lipid content was achieved by *P. kudriavzevii* V194—64 % (*w*/*w*), lipid yield of 0.41 g g^−1^ C (grams per gram of carbon consumed), or 0.17 g g^−1^ of acetic acid consumed, corresponding to a lipid output of 2.5 g L^−1^ (all differences are statistically significant at *p* < 0.01 in comparison with other yeasts). Considering that the maximum theoretical yield of lipids produced per acetic acid consumed is estimated to be 0.27 g g^−1^ [47], *P. kudriavzevii* V194 produced 63% of the theoretical lipid value. *A. brassicae* V134 also produced more than 50% (*w*/*w*) of lipids, but for the remaining two species, this value was reduced to half. Although *A. brassicae* V134 presented high percentage of lipids content, the lipid was only of 0.09 g g ^−1^ of acetic acid consumed, due to the lower biomass production. Some authors recommend concentrations of VFAs below 5 g L^−1^ to avoid an acid inhibitory effect [19,48]. Possibly, the low lipid yield of 0.09 g g^−1^ of acetic acid consumed presented by *A. brassicae* V134 is due to acid inhibitory effect.

Long-chain fatty acid composition is presented in Table 3 and revealed some differences between the four isolates. For strains *A. brassicae* V134 and *P. kudriavzevii* V194, about 50% of the LCFAs synthesized were unsaturated, oleic (C18:1n9) acid being the predominant one. Palmitic (C16:0) and stearic (C18:0) acids were the main saturated fatty acids produced by the two isolates. For strains *C. tropicalis* V139 and *M. pulcherrima* V213, the lipid composition was also identical but different from that of *A. brassicae* V134 and *P. kudriavzevii* V194. For those strains, palmitic and stearic saturated acids corresponded to approximately 25% of the total LCFAs measured, half of what was found in *A. brassicae* V134 and *P. kudriavzevii* V194. The unsaturated form, distributed mainly by oleic (ca. 60%), palmitoleic (ca. 10%), and linoleic (ca. 5%) acids, represented about 75% of the total LCFAs characterized. Palmitoleic acid was synthesized by *C. tropicalis* V139 and *M. pulcherrima* V213 (ca. 10%) and was not detected in *A. brassicae* V134 and *P. kudriavzevii* V194. The LCFAs profiles variation observed between yeasts could, certainly, influence the selection process, in addition to their production capabilities, according to the goal of the industrial applications.

### 3.2. Effect of Different VFAs on Biomass Growth and SCOs Production

**Figure 2:**
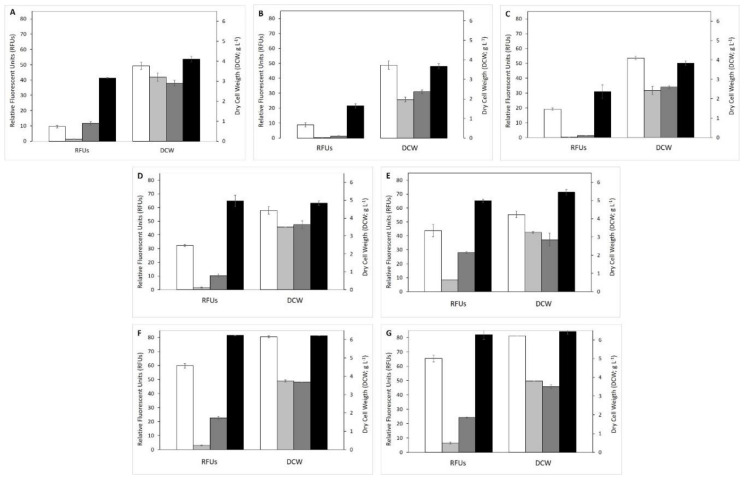
Effect of volatile fatty acids (pure or in mixture) on single cell oil production (relative fluorescent units, RFUs) and yeast growth (dry cell weight, DCW) using the four tested yeast species: white—*Apiotrichum brassicae* V134; light grey—*Candida tropicalis* V139; dark grey—*Metschnikowia pulcherrima* V213; black—*Pichia kudriavzevii* V194. Letters indicate the medium in which yeasts were grown, during 120 h: (**A**) YP:HAc (5.1 g L^−1^); (**B**) YP:HPr (2.7 g L^−1^); (**C**) YP:HBut (3.5 g L^−1^); (**D**) YP:HAc:HPr; (**E**) YP:HAc:HBut; (**F**) YP:HAc:HPr:HBut; (**G**) YP:HAc:HPr:HBut:isoHbut (0.2 g L^−1^):HVal (0.9 g L^−1^). Values represent mean of triplicates ± standard deviations.

After time course analysis and lipid production characterization in medium with acetic acid as the sole carbon source, the effect of different VFAs on yeast growth and lipid production was explored. With this approach, we intended to improve the knowledge on the effect of different VFAs when used as carbon sources, individually or in different combinations, since information in the literature is still scarce. For this, experiments were conducted in YP medium supplemented with different types and concentrations of VFAs (Table 1) for 120 h. Results of biomass (DCW) and SCOs production (RFUs) of the four oleaginous yeasts are presented in Figure 2, obtained when testing the individual effect of HAc (Figure 2A), HPr (Figure 2B), and HBut (Figure 2C), together with the effect of mixtures of HAc:HPr (Figure 2D), Hac:HBut (Figure 2E), HAc:HBut:HPr (Figure 2F), and HAc:HBut:HPr:isoHBut:isoHVal (Figure 2G), at similar concentrations as the ones found in the effluent filtrate (Table 1).

#### 3.2.1. Individual VFAs Effect in Biomass and Lipid Production

The four yeasts were able to grow in the three individual VFAs, although *A. brassicae* V134 and *P. kudriavzevii* V194 revealed higher biomass production and higher lipid accumulation for HPr and HBut in comparison with *C. tropicalis* V139 and *M. pulcherrima* V213. A different result was observed for growth in acetic acid (5.1 g L^−1^), at which almost no lipid accumulation was detected for *C. tropicalis* V139 (1.4 ± 0.2 RFUs), identical lipid content was obtained for *A. brassicae* V134 (10 ± 1 RFUs) and *M. pulcherrima* V213 (12 ± 1 RFUs), and the highest accumulation was observed for *P. kudriavzevii* V194 (41.2 ± 0.2 RFUs; *p* < 0.01). The latter produced about 75% more RFUs than strains *A. brassicae* V134 and *M. pulcherrima* V213, and 96% more than strain *C. tropicalis* V139. Results suggest that the four yeasts grow well (shown by high DCW values), having individual VFAs as carbon sources, but not all could be considered good SCOs producers (low RFUs values).

The observed differences between yeast isolates can stem from their individual metabolism, allowing them to metabolize or not the aforementioned VFAs into lipids. *A. brassicae* V134 showed to be a good SCOs producer in all the carbon sources tested. However, if the results of fluorescence are normalized per gram of carbon initially added (RFU g^−1^ C_added_; Figure S2), acetic acid was the carbon source that leads to the lowest amount of lipids when compared to propionic and butyric acids. That result was unexpected, considering that acetic acid was the VFA whose metabolism is described as the simplest, and, usually, inhibitory effects increase with the VFA’s chain length [16]. In fact, for this yeast isolate, butyric acid was the carboxylic acid that yielded higher DCW and lipid accumulation, supporting the role of carbon source in growth and lipid production. Additionally, it showed that some microorganisms could have different VFAs preferences, probably according to the enzymes they synthesize for VFAs uptake. The same possibility has already been hypothesized by Fradinho et al. [49]. Another possibility is related to the conclusion that concentrations of VFAs should be below 5 g L^−1^ to avoid acid inhibitory effect [19,48]. However, when subjected to a medium with 15 g L^−1^ of acetic acid (Section 3.1), our results show that this yeast grew well and produced a high amount of SCOs, without an apparent inhibitory effect.

*P. kudriavzevii* V194 was the yeast strain that accumulated a higher amount of lipids in all individual VFAs tested. The highest accumulation of neutral lipids occurred when acetic acid was the carbon source, corroborating what is described in the literature [13], and already discussed. When butyric and propionic acids were used as carbon sources, the fluorescence signal was reduced, indicating that fewer neutral lipids were synthesized. However, yeast growth was also lower, indicating that these carbon sources were not being used for the cellular growth process. Deeper studies are necessary to understand the role of the two acids in the cellular biological processes associated with this species.

One important factor to take into account is the divergent initial carbon sources concentrations, lower for some of the tested VFAs, but justifiable since we want to mimic the values found in the effluent filtrate. This discrepancy could lead to distinct carbon/nitrogen ratios, and, consequently, to dissimilar results, since lipid accumulation is triggered when nitrogen is depleted from the medium [5]. In this way, we normalized the fluorescence results per gram of carbon initially added to each medium (RFU g^−1^ C) in order to compare between different media. Results are shown in Figure S2 and one can conclude that acetic acid was the carbon source that individually had higher outcomes in lipid accumulation for the yeasts *C. tropicalis* V139, *P. kudriavzevii* V194, and *M. pulcherrima* V213, whereas butyric acid had the lowest. Curiously, strain *A. brassicae* V134 had an opposite preference: butyric acid was the carbon source that led to higher values of lipid accumulation, followed by propionic and acetic acids.

#### 3.2.2. Effect of VFAs Mixtures in Biomass and Lipid Production

After analyzing the individual effects of the three VFAs, mixtures of two, three, and five synthetic VFAs were tested.

In medium with a mixture of HAc:HPr (Figure 2D), all strains produced SCOs, although *C. tropicalis* V139 production was significantly different (*p* < 0.01) and almost negligible. *P. kudriavzevii* V194 was again the isolate with higher RFUs detected, a value 50% and 94% higher than the ones obtained by isolates *C. tropicalis* V139 and *M. pulcherrima* V213, respectively (*p* < 0.01). When testing the mixture of HAc:HBut (Figure 2E), all strains obtained considerable amounts of RFUs, the differences not being as pronounced (although still significant for all strains) as in the previous mixture: yeast *A. brassicae* V134 had 33% fewer RFUs than *P. kudriavzevii* V194, and strain *M. pulcherrima* V213 about 57% fewer lipids than strain *P. kudriavzevii* V194. These results seem to indicate a synergistic effect between acetic and butyric acids, considering that the lipid accumulation was superior when both were used.

When a combination of the three main VFAs (HAc: HPr: HBut) was tested as the carbon source for yeast growth (Figure 2F), the pattern of RFUs registered was similar to the one obtained in the mixtures of only two acids: yeast *P. kudriavevii* V194 showed, again, superior lipid production, being 96% higher than the yeast with lower production—*C. tropicallis* V139 (*p* < 0.01). In fact, with regard to yeast *C. tropicalis* V139, and especially concerning its biomass production and lipid synthesis (Figure 2F), the quantified RFUs were not high, although the biomass production was acceptable, suggesting that the carbon sources used were directed to growth instead of lipid accumulation. This result is in accordance with the one reported by Arous et al. [50], in which different *Candida* species had a reasonable biomass production (5.6 to 8.9 g L^−1^), despite the percentage of lipids accumulated being low (8.5 to 12.9% *w*/*w*). Likewise, in the study of Ayadi et al. [10], *C. viswanathii* had a lipid content of only 25% (*w*/*w*) and a biomass production of 13 g L^−1^. For the isolate *M. pulcherrima* V213, the mixture of HAc:HBut showed a synergistic effect and was the combination that induced higher lipidic accumulations, an observation concordant with the ones of Fradinho et al. [49], which showed that acetic acid could act as a co-substrate for butyric uptake. When propionic acid was added to the mixture of acetic and butyric acids, the lipid production fell 63% for *C. tropicalis* V139 and 20% for *M. pulcherrima* V213, which means it has an inhibitory effect in lipid production. However, for *A. brassicae* V134 and *P. kudriavevii* V194, the addition of propionic acid resulted in higher RFUs values than the ones obtained for the mixture of the two acids.

Results similar to the ones obtained with the three VFAs mixture were obtained when testing the combination of five VFAs (HAc:HPr:HBut:isoHBut:HVal; Figure 2G). These results seem to indicate that, at these concentrations (Table 1), neither isobutyric nor valeric acids contribute significantly to lipid synthesis in the conditions tested except for *C. tropicalis* V139. This strain presented twice the RFUs values obtained with the mixture of HAc:HPr:HBut (see also Figure S2), revealing a metabolism very distinct from the other three strains and suggesting that valeric and isobutyric acids stimulated lipids synthesis.

In this way, and considering the overall results, the combination of the three main VFAs—acetic, propionic, and butyric acids—seems to be the best option to achieve high lipid accumulation for the majority of the yeasts.

These variable results support the conclusion that many factors influence lipid production by yeasts, not only yeast species and strains’ physiological abilities, but also substrate type, growth conditions, C/N ratio, etc. [5,51,52].

### 3.3. Effluent Filtrate as Fermentation Medium

The effluent filtrate obtained from the anaerobic digestion of organic wastes was used as a suitable medium for biomass and lipid production. The same effluent was used before to assess the growth of *Crypthecodinium cohnii*, a microalgae, that was able to deplete the organic acid content of the effluent after 60 h of fed-batch cultivation [53].

Our previous results brought insights into the growth processes and lipid accumulation, VFAs preferences, and LCFAs profile for each of the selected yeast strains, in synthetic VFAs media. However, when using yeasts to ferment unpurified VFAs present in a real effluent, one has to deal with its complex composition and its inhibitory effects. In this way, growth experiments were conducted in shake flasks for 120 h in the effluent filtrate. The main VFAs present in the effluent filtrate, as detailed in Table 1, were: acetic, propionic, and butyric acids and, in smaller amounts (less than 1 g L^−1^), isobutyric, valeric, isovaleric, and caproic acids, at pH 7.0. As shown in Table 2, from the four yeasts studied, two stand out for all the parameters: *A. brassicae* V134 and *P. kudriavzevii* V194. *A. brassicae* V134 and *P. kudriavzevii* V194 obtained higher biomass productions (8.3 ± 0.1 and 7.8 ± 0.4 g L^−1^, respectively) and accumulated a higher percentage of lipids (43 ± 3 and 47 ± 2 % (*w*/*w*), respectively), these productions being significantly higher than the ones obtained by the other two isolates (*p* < 0.01). As a consequence of high biomass and lipid production, both the strains *A. brassicae* V134 and *P. kudriavzevii* V194 had a lipid yield of ca. 0.5 g g^−1^ C_consumed_ (0.25 g g^−1^ VFA_consumed_). When comparing to other studies, these values are twice the maximum lipid yield of 0.13 g g^−1^ VFA_consumed_ reported by Llamas et al. [19] using an anaerobic digestion effluent, but with other yeast species: *Cutaneotrichosporon curvatum.* Similar lipid yield values were obtained for *Rhodosporidium toruloides* (0.23 g g^−1^; [54]), *Yarrowia lipolytica* (0.27 g g^−1^; [55]), and *Mortierella isabelline* (0.25 g g^−1^, [23]), using glucose as the carbon source. According to Kim et al. [47], the maximum theoretical lipid yield for different VFA consumed are: 0.27 g g^−1^ for acetic acid, 0.38 g g^−1^ for propionic acid, 0.47 g g^−1^ for butyric acid, 0.52 g g^−1^ for valeric acid, and 0.57 g g^−1^ for caproic acid. Considering the composition and consumption of each VFA in our effluent filtrate, the maximum theoretical yield calculated in our conditions would be ~0.39 g g^−1^ of VFA_consumed_. Thus, we obtained 68% efficiency in VFA conversion into lipids for both *A. brassicae* V134 and *P. kudriavzevii* V194. We consider this to be a very good result, considering that the maximum efficiency in glucose conversion was 78%, obtained in shake flasks for *Mortierella isabellina* [23].

The anaerobic digestion effluent with 15 g L^−1^ of VFAs used by Llamas et al. [19] is identical in VFAs composition and concentrations to the effluent filtrate used in our study but differs in nitrogen concentration, leading to different C/N. Therefore, we attribute the high lipid yield achieved in our study to the higher C/N found in the effluent filtrate and, eventually, to its very small amount of nitrogen available (0.9 g N L^−1^). Curiously, for these yeasts, after 120 h of growth, the color of the medium changed from white to brown. Although we could not determine the nature of this phenomenon, we can only speculate that some biochemical or physicochemical process occurred due to the pH increase from 7.0 to 8.7. In comparison with the lower value (20.34%) associated with *A. brassicae* in the literature [28], even despite the different conditions, it is possible to conclude that the oleaginous character is strain-dependent and our strain is more suitable for SCOs production. In the future, it would be interesting to optimize the growth and culture conditions to increase its lipid productivity and biomass accumulation.

**Figure 3:**
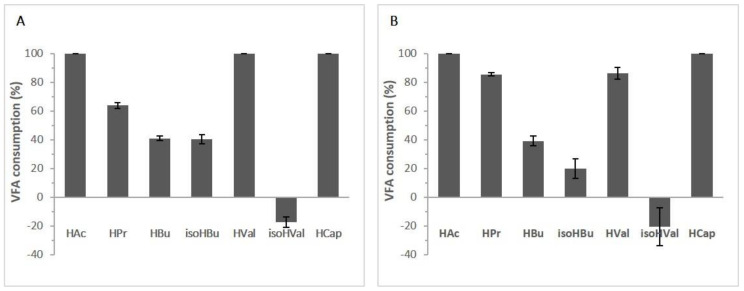
Volatile fatty acid (VFA) consumption (%) after 120 h of incubation in effluent filtrate obtained from anaerobic digestion of organic wastes for: (**A**) *Candida tropicalis* V139 and (**B**) *Metschnikowia pulcherrima* V213. Values represent mean of triplicates ± standard deviations.

Oppositely, strains *C. tropicalis* V139 and *M. pulcherrima* V213 showed biomass (6.6 ± 0.2 and 6.5 ± 0.4 g L^−1^, respectively) and lipid (20 ± 2 and 24 ± 2%, respectively) productions not as high as expected, and smaller lipid yields (0.29 and 0.33 g g^−1^ C_consumed_ or 0.14 and 0.16 g g^−1^ VFA_consumed_, respectively). Since the low lipid synthesis by these two isolates could be attributed to inefficient metabolism of the carbon sources available, we analyzed the profile of VFAs consumption after fermentation (Figure 3). All VFAs available in the effluent filtrate were totally consumed by isolate *A. brassicae* V134 (100%) and *P. kudriavzevii* V194 (97–100%), but a variable consumption pattern associated to *C. tropicalis* V139 and *M. pulcherrima* V213 was observed. *C. tropicalis* V139 consumed 100% of the acetic, valeric, and caproic acids, but less than 50% of the butyric and isobutyric acids. Strain *M. pulcherrima* V213 only consumed the acetic and caproic acids completely, butyric and isobutyric acids being less than one-third degraded. An unforeseen result was the production of isovaleric acid by *C. tropicalis* V139 and *M. pulcherrima* V213, which could be owed to isomerism reactions. Considering the results obtained when using the effluent filtrate as fermentation medium, yeasts *A. brassicae* V134 and *P. kudriavzevii* V194 appeared to be the most suitable strains to be used for SCOs production at a larger scale, owing to their growth and lipid accumulation abilities.

The long-chain fatty acids profile, being an essential point required for a profitable and economic process of SCOs production, was evaluated. Globally, oleic (C18:1n9), palmitic (C16:0), stearic (C18:0), and cis-10-heptadecenoic (C17:1) acids were the main LCFAs synthesized. As shown in Table 3, all the strains produced high percentages of oleic acid (ca. 42%), the main component of olive oil. *A. brassicae* V134 and *P. kudriavzevii* V194 had a similar pattern of LCFAs produced. Besides oleic acid, *A. brassicae* V134 and *P. kudriavzevii* V194 synthesized ca. 20% (*v*/*v*) of both palmitic (C16:0) and stearic (C18:0) acids, ca. 5% (*v*/*v*) of both linoleic (C18:2n6, an omega-6 fatty acid) and cis-10-heptadecenoic (C17:1) acids, 3% (*v*/*v*) of eicosenoic (C20:1n9) acid, and 1.5% (*v*/*v*) of pentadecanoic (C15:0) acid. Strains *C. tropicalis* V139 and *M. pulcherrima* V213 also presented a similar pattern of LCFAs synthesized, with about 70% of unsaturated LCFAs, mainly oleic (C18:1n9, ca. 42% (*v*/*v*)) and cis-10-heptadecenoic (C17:1, 15.5–22% (*v*/*v*)) acids and also ca. 10% (*v*/*v*) of both palmitic (C16:0) and stearic (C18:0) acids, 3–10% (*v*/*v*) of linoleic (C18:2n6, an omega-6 fatty acid), and less than 3% (*v*/*v*) of the three pentadecanoic (C15:0), palmitoleic (C16:1), and eicosenoic (C20:1n9) acids. The LCFAs in Table 3 are those that presented concentrations higher than 1% (*v*/*v*) but other fatty acids were detected at concentrations below 1% (*v*/*v*; data not shown) and may be important to report. Thus, LCFA as γ-linolenic (C18:3n6, an omega-6 fatty acid) acid was detected for the four yeasts. While *A. brassicae* V134 and *M. pulcherrima* V213 had ca. 1% (*v*/*v*), for the yeasts *C. tropicalis* V139 and *P. kudriavzevii* V194 less than 1% was detected; therefore, they are not shown in Table 3. Capric (C10:0), undecanoic (C11:0), palmitoleic (C16:1), and cis-11-eicosenoic (C20:1n9) acids were detected for *C. tropicalis* V139 and *M. pulcherrima* V213; myristic acid (C14:0) for *A. brassicae* V134, *C. tropicalis* V139, and *P. kudriavzevii* V194; α-linolenic acid (C18:3n3, an omega-3 fatty acid) for *A. brassicae* V134, *P. kudriavzevii* V194, and *M. pulcherrima* V213. As can be noted, some essential fatty acids were synthesized, namely omega-3 and omega-6 fatty acids.

Regarding strain *P. kudriavzevii* V194’s profile, a more uniform distribution between saturated and unsaturated fatty acids was obtained, with 44% being saturated and 56% unsaturated. The distribution among the fatty acids was similar to strain *A. brassicae* V134, with a slight increase in the production of the omega-3 and omega-6 fatty acids. Globally, the results are surprising, especially regarding *A. brassicae* V134 and *P. kudriavzevii* V194. It is reported that microbial lipids synthesized using propionic acid as a carbon source would contain more odd-numbered fatty acids [18]. In our work, when growing in the effluent filtrate, these two yeasts were capable of completely consuming propionic acid (Figure 3). However, the content of pentadecanoic (C15:0) and cis-10-heptadecenoic acids (C17:1) was lower in these strains when compared with *C. tropicalis* V139 and *M. pulcherrima* V213, going against what was stated.

In recent years, the use of effluents as feedstocks for microbial lipid production gained increased importance, as already referred, since, from an economic standpoint, the use of inexpensive carbon sources to synthesize added value fatty acids is desired. However, reports exploring this topic are still limited [16,19,56]. Our main result showed that pentadecanoic (C15:0), cis-10-heptadecenoic acids (C17:1), γ-linoleic (C18:2n6, an omega-6 fatty acid), and cis-11-eicosenoic (C20:1n9) fatty acids were only synthesized in fermentations using the effluent filtrate.

The overall fatty acid composition obtained with yeasts *A. brassicae* V134 and *P. kudriavzevii* V194 was not significantly different between media. However, for yeasts *C. tropicalis* V139 and *M. pulcherrima* V213, differences in the LCFAs profile were noteworthy, with about 20% fewer oleic acid synthesized in the effluent filtrate when compared with synthetic medium YP:HAc (15 g L^−1^). The type of LCFAs produced is in accordance with what is described in the literature, and the amounts obtained for oleic (42–44%), palmitic (8–21%), and linoleic (3–10%) acids revealed high similarity with vegetable oils used in industrial applications [19,57], in particular in biofuel production and manufacturing of cosmetics (palmitic and oleic acids), in pharmaceutical applications (oleic acid), and as antioxidants (linoleic acid) (reviewed in [58]).

## 4. Conclusions

In conclusion, our results support and validate the effluent filtrate obtained through anaerobic digestion of organic wastes as a suitable medium for yeast growth and single cell oil production. All the used yeast strains were able to grow and produce valuable long-chain fatty acids when grown in the dark fermentation effluent filtrate, although lipid outputs of strains V139 and V213 were low. In view of the results, we propose strains *A. brassicae* (V134) and *P. kudriavzevii* (V194) as potential candidates for SCOs production at larger scales through the use of an inexpensive carbon source, combining economic and environmental benefits.

## Figures and Tables

**Table 1:** Media composition (g L^−1^) and characteristics.

	YP:HAc	YP:HPr	YP:HBut	YP:HAc:HPr	YP:HAc:HBut	YP:HAc:HPr:HBut	YP:HAc:HPr:HBut:isoHBut:HVal	Effluent Filtrate
Yeast extract	5	5	5	5	5	5	5	-
Peptone	10	10	10	10	10	10	10	-
HAc	15/5.1	-	-	5.1	5.1	5.1	5.1	5.1
HPr	-	2.7	-	2.7	-	2.7	2.7	2.7
HBut	-	-	3.5	-	3.5	3.5	3.5	3.5
isoHBut	-	-	-	-	-	-	0.2	0.2
HVal	-	-	-	-	-	-	0.9	0.9
isoHVal	-	-	-	-	-	-	-	0.3
HCap	-	-	-	-	-	-	-	1.0
Total dissolved organic carbon	13.6/9.7	9.0	9.5	11.0	11.6	12.9	13.5	6.9
Total dissolved nitrogen	3.2	3.2	3.2	3.2	3.2	3.2	3.2	0.9
Carbon/Nitrogen	4.3/3.0	2.8	3.0	3.4	3.6	4.0	4.2	7.6
pH	6.9	6.9	6.9	6.9	6.9	6.9	6.9	7.0

HAc—acetic acid; HPr—propionic acid; HBut—butyric acid; isoHBut—isobutyric acid; HVal—valeric acid; isoHVal—isovaleric acid; HCap—caproic acid.

**Table 2:** Biomass, lipid production, and carbon source utilization by the four selected yeast isolates in YP:HAc 15 g L^−1^ and in effluent filtrate, after 120 h of cultivation.

Medium	Yeasts	Dry Cell Weight (g L^−1^)	Lipid Content (%, *w*/*w*)	Lipid Output (g L^−1^)	Y_X/S_ (g g^−1^ C_consumed_)	Y_L/S_ (g g^−1^ C_consumed_)	Y_L/S_ (g g^−1^ VFA_consumed_)	Carbon Source Reduction (%)
*YP:HAc 15 g L^−1^*	*A. brassicae* V134	2.5 ± 0.2	55 ± 2	1.4 ± 0.2	0.41 ± 0.04	0.23 ± 0.02	0.09 ± 0.01	100
	*C. tropicalis* V139	3.56 ± 0.03	27 ± 1	0.92 ± 0.05	0.60 ± 0.01	0.15 ± 0.01	0.062 ± 0.004	100
	*M. pulcherrima* V213	3.5 ± 0.3	29 ± 1	1.03 ± 0.03	0.59 ± 0.01	0.17 ± 0.01	0.070 ± 0.002	100
	*P. kudriavzevii* V194	3.86 ± 0.04	64 ± 4	2.5 ± 0.1	0.65 ± 0.01	0.41 ± 0.02	0.17 ± 0.01	100
*Effluent filtrate*	*A. brassicae* V134	8.3 ± 0.1	43.2 ± 3	3.40 ± 0.1	1.24 ± 0.02	0.51 ± 0.02	0.25 ± 0.01	100
	*C. tropicalis* V139	6.6 ± 0.2	19.6 ± 2	1.38 ± 0.2	1.36 ± 0.03	0.29 ± 0.04	0.14 ± 0.02	72
	*M. pulcherrima* V213	6.5 ± 0.4	23.6 ± 2	1.65 ± 0.1	1.31 ± 0.08	0.33 ± 0.02	0.16 ± 0.01	74
	*P. kudriavzevii* V194	7.8 ± 0.4	46.7 ± 2	3.33 ± 0.07	1.17 ± 0.06	0.50 ± 0.01	0.25 ± 0.01	99

Y_X/S_—growth yield (g DCW per g carbon consumed); Y_L/S_—lipid yield (g lipids per g carbon or g VFA consumed). Results represent mean of triplicates ± standard deviations.

**Table 3:** Long-chain fatty acid profiles (%) obtained for the four yeast isolates after 120 h of growth in YP:HAc 15 g L^−1^ and in the effluent filtrate. Results represent mean of triplicates ± standard deviations.

		C14:0	C15:0	C16:0	C16:1	C17:1	C18:0	C18:1n9	C18:2n6	C18:3n6	C20:1n9	Sum
		Myristic Acid	Pentadecanoic Acid	Palmitic Acid	Palmitoleic Acid	Heptadecenoic Acid	Stearic Acid	Oleic Acid	Linoleic Acid	γ-Linolenic Acid	Eicosenoic Acid	
*YP:HAc 15 g L^−1^*	*A. brassicae* V134	1.05 ± 0.01		26.8 ± 0.3			19.5 ± 0.1	47.5 ± 0.4	2.4 ± 0.1			97
	*C. tropicalis* V139			13.9 ± 0.4	9.7 ± 0.4		8.7 ± 0.5	61.8 ± 0.3	4.2 ± 0.4			98
	*M. pulcherrima* V213			15.4 ± 0.4	9.2 ± 0.2		10.0 ± 0.6	60.0 ± 0.8	3.4 ± 0.1			98
	*P. kudriavzevii* V194			25.6 ± 0.4			18.5 ± 0.6	50.3 ± 0.1	2.8 ± 0.1			97
*Effluent filtrate*	*A. brassicae* V134		1.5 ± 0.1	19.5 ± 0.1		5.2 ± 0.5	19.9 ± 0.1	44 ± 0.4	4 ± 2	1.2 ± 0.2	3.1 ± 0.3	98
	*C. tropicalis* V139		2.1 ± 0.1	10.2 ± 0.3	2.9 ± 0.1	15.5 ± 0.6	11.8 ± 0.3	41.5 ± 0.2	9.5 ± 0.4		2.6 ± 0.1	96
	*M. pulcherrima* V213		2.0 ± 0.5	7.7 ± 0.1	2.4 ± 0.1	22 ± 6	12.0 ± 0.3	42 ± 8	3.1 ± 0.4	1.0 ± 0.2	3.4 ± 0.8	95
	*P. kudriavzevii* V194		1.6 ± 0.1	20.7 ± 0.2		4.5 ± 0.1	19.9 ± 0.2	42.3 ± 0.3	4.9 ± 0.3		2.5 ± 0.1	96

## Supplementary Materials

The following are available online at https://www.mdpi.com/2076-2607/8/11/1809/s1, Figure S1: Time-course growth in YP:HAc (15g L^−1^) medium, during 120 h, at pH 5.5, Figure S2: Lipid production (relative fluorescent units, RFUs) and growth (OD_640_) normalized per gram of carbon initially added in each medium, after 120 h of growth, Table S1: Biomass, lipid production and carbon source utilization in medium containing acetic acid (15 g L^−1^) as carbon source, at pH 5.5, Table S2: Long chain fatty acids profiles (%) obtained after 120 h of growth in medium containing acetic acid (15 g L^−1^), at pH 5.5.

## References

[1] De Jong E., Jungmeier G. (2015). Biorefinery Concepts in Comparison to Petrochemical Refineries.

[2] Adrio J.L. (2017). Oleaginous yeasts: Promising platforms for the production of oleochemicals and biofuels. Biotechnol. Bioeng..

[3] Metzger J.O., Bornscheuer U. (2006). Lipids as renewable resources: Current state of chemical and biotechnological conversion and diversification. Appl. Microbiol. Biotechnol..

[4] Xue S.J., Chi Z., Zhang Y., Li Y.F., Liu G.L., Jiang H., Hu Z., Chi Z.M. (2018). Fatty acids from oleaginous yeasts and yeast-like fungi and their potential applications. Crit. Rev. Biotechnol..

[5] Sitepu I.R., Garay L.A., Sestric R., Levin D., Block D.E., German J.B., Boundy-Mills K.L. (2014). Oleaginous yeasts for biodiesel: Current and future trends in biology and production. Biotechnol. Adv..

[6] Thorpe R.F., Ratledge C. (1972). Acid Distribution in Triglycerides. J. Gen. Microbiol..

[7] Ageitos J.M., Vallejo J.A., Veiga-Crespo P., Villa T.G. (2011). Oily yeasts as oleaginous cell factories. Appl. Microbiol. Biotechnol..

[8] Miranda C., Bettencourt S., Pozdniakova T., Pereira J., Sampaio P., Franco-Duarte R., Pais C. (2020). Modified high-throughput Nile red fluorescence assay for the rapid screening of oleaginous yeasts using acetic acid as carbon source. BMC Microbiol..

[9] Garay L.A., Sitepu I.R., Cajka T., Chandra I., Shi S., Lin T., German J.B., Fiehn O., Boundy-Mills K.L. (2016). Eighteen new oleaginous yeast species. J. Ind. Microbiol. Biotechnol..

[10] Ayadi I., Kamoun O., Trigui-Lahiani H., Hdiji A., Gargouri A., Belghith H., Guerfali M. (2016). Single cell oil production from a newly isolated Candida viswanathii Y-E4 and agro-industrial by-products valorization. J. Ind. Microbiol. Biotechnol..

[11] Arous F., Frikha F., Triantaphyllidou I.E., Aggelis G., Nasri M., Mechichi T. (2016). Potential utilization of agro-industrial wastewaters for lipid production by the oleaginous yeast Debaryomyces etchellsii. J. Clean. Prod..

[12] Dourou M., Kancelista A., Juszczyk P., Sarris D., Bellou S., Triantaphyllidou I.E., Rywinska A., Papanikolaou S., Aggelis G. (2016). Bioconversion of olive mill wastewater into high-added value products. J. Clean. Prod..

[13] Park Y.K., Dulermo T., Ledesma-Amaro R., Nicaud J.M. (2018). Optimization of odd chain fatty acid production by Yarrowia lipolytica. Biotechnol. Biofuels.

[14] Jiang J., Zhang Y., Li K., Wang Q., Gong C., Li M. (2013). Volatile fatty acids production from food waste: Effects of pH, temperature, and organic loading rate. Bioresour. Technol..

[15] Hoornweg D., Bhada-Tata P. (2012). What a Waste: A Global Review of Solid Waste Management—Review, Global Management, Solid Waste.

[16] Gao R., Li Z., Zhou X., Cheng S., Zheng L. (2017). Oleaginous yeast Yarrowia lipolytica culture with synthetic and food waste-derived volatile fatty acids for lipid production. Biotechnol. Biofuels.

[17] Fontanille P., Kumar V., Christophe G., Nouaille R., Larroche C. (2012). Bioconversion of volatile fatty acids into lipids by the oleaginous yeast Yarrowia lipolytica. Bioresour. Technol..

[18] Kolouchová I., Schreiberová O., Sigler K., Masák J., Řezanka T. (2015). Biotransformation of volatile fatty acids by oleaginous and non-oleaginous yeast species. FEMS Yeast Res..

[19] Llamas M., Dourou M., González-Fernández C., Aggelis G., Tomás-Pejó E. (2020). Screening of oleaginous yeasts for lipid production using volatile fatty acids as substrate. Biomass Bioenergy.

[20] Gientka I., Gadaszewska M., Błażejak S., Kieliszek M., Bzducha-Wróbel A., Stasiak-Różańska L., Kot A.M. (2017). Evaluation of lipid biosynthesis ability by Rhodotorula and Sporobolomyces strains in medium with glycerol. Eur. Food Res. Technol..

[21] Ratledge C. (2004). Fatty acid biosynthesis in microorganisms being used for Single Cell Oil production. Biochimie.

[22] Niehus X., Casas-Godoy L., Vargas-Sánchez M., Sandoval G. (2018). A Fast and Simple Qualitative Method for Screening Oleaginous Yeasts on Agar. J. Lipids.

[23] Papanikolaou S., Aggelis G. (2019). Sources of microbial oils with emphasis to Mortierella (Umbelopsis) isabellina fungus. World J. Microbiol. Biotechnol..

[24] Bellou S., Triantaphyllidou I.E., Aggeli D., Elazzazy A.M., Baeshen M.N., Aggelis G. (2016). Microbial oils as food additives: Recent approaches for improving microbial oil production and its polyunsaturated fatty acid content. Curr. Opin. Biotechnol..

[25] Ratledge C. (2013). Microbial oils: An introductory overview of current status and future prospects. OCL—Oilseeds fats. Crop. Lipids.

[26] Fillet S., Ronchel C., Callejo C., Fajardo M.J., Moralejo H., Adrio J.L. (2017). Engineering Rhodosporidium toruloides for the production of very long-chain monounsaturated fatty acid-rich oils. Appl. Microbiol. Biotechnol..

[27] Wu J., Zhang X., Xia X., Dong M. (2017). A systematic optimization of medium chain fatty acid biosynthesis via the reverse beta-oxidation cycle in *Escherichia coli*. Metab. Eng..

[28] Franklin S., Decker S.M., Wee J. (2011). United States Patent: Fuel and Chemical Production from Oleaginous Yeast 2011. U.S. Patent.

[29] Hassan M., Blanc P.J., Granger L.M., Pareilleux A., Goma G. (1993). Lipid production by an unsaturated fatty acid auxotroph of the oleaginous yeast Apiotrichum curvatum grown in single-stage continuous culture. Appl. Microbiol. Biotechnol..

[30] Ykema A., Kater M.M., Smit H. (1989). Lipid production in wheypermeate by an unsaturated fatty acid mutant of the oleaginous yeast Apiotrichum curvatum. Biotechnol. Lett..

[31] Heredia L., Ratledge C. (1988). Simultaneous utilization of glucose and xylose by Candida curvata D in continuous culture. Biotechnol. Lett..

[32] Solaiman D.K.Y., Ashby R.D., Nuñez A., Foglia T.A. (2004). Production of sophorolipids by Candida bombicola grown on soy molasses as substrate. Biotechnol. Lett..

[33] Dey P., Maiti M.K. (2013). Molecular characterization of a novel isolate of Candida tropicalis for enhanced lipid production. J. Appl. Microbiol..

[34] Louhasakul Y., Cheirsilp B., Maneerat S., Prasertsan P. (2019). Potential use of flocculating oleaginous yeasts for bioconversion of industrial wastes into biodiesel feedstocks. Renew. Energy.

[35] Polburee P., Yongmanitchai W., Lertwattanasakul N., Ohashi T., Fujiyama K., Limtong S. (2015). Characterization of oleaginous yeasts accumulating high levels of lipid when cultivated in glycerol and their potential for lipid production from biodiesel-derived crude glycerol. Fungal Biol..

[36] Zhang X., Yan S., Tyagi R.D., Surampalli R.Y., Valéro J.R. (2014). Wastewater sludge as raw material for microbial oils production. Appl. Energy.

[37] Rangaswamy V., Saran S., Kannabiran M., Thiru M., Sankh S. (2017). Bioprocess for Biodiesel Production from a Yeast Strain 2017. U.S. Patent.

[38] Sankh S., Thiru M., Saran S., Rangaswamy V. (2013). Biodiesel production from a newly isolated Pichia kudriavzevii strain. Fuel.

[39] Woodbine M. (1959). Microbial fat: Microorganisms as potential fat producers. Prog. Ind. Microbiol..

[40] Sitepu I.R., Ignatia L., Franz A.K., Wong D.M., Faulina S.A., Tsui M., Kanti A., Boundy-Mills K. (2012). An improved high-throughput Nile red fluorescence assay for estimating intracellular lipids in a variety of yeast species. J. Microbiol. Methods.

[41] Dourou M., Mizerakis P., Papanikolaou S., Aggelis G. (2017). Storage lipid and polysaccharide metabolism in Yarrowia lipolytica and Umbelopsis isabellina. Appl. Microbiol. Biotechnol..

[42] Beopoulos A., Nicaud J.M. (2012). Yeast: A new oil producer? OCL—Ol. Corps Gras Lipides.

[43] Papanikolaou S., Chevalot I., Komaitis M., Aggelis G., Marc I. (2001). Kinetic profile of the cellular lipid composition in an oleaginous Yarrowia lipolytica capable of producing a cocoa-butter substitute from industrial fats. Antonie Van Leeuwenhoek.

[44] Carsanba E., Papanikolaou S., Fickers P., Erten H. (2020). Lipids by yarrowia lipolytica strains cultivated on glucose in batch cultures. Microorganisms.

[45] Xenopoulos E., Giannikakis I., Chatzifragkou A., Koutinas A., Papanikolaou S. (2020). Lipid production by yeasts growing on commercial xylose in submerged cultures with process water being partially replaced by olive millwastewaters. Processes.

[46] Serrano-Carreon L., Hathout Y., Bensoussan M., Belin J. (1992). Lipid accumulation in Trichoderma species. FEMS Microbiol. Lett..

[47] Kim N.-J., Lim S.-J., Chang H.N. (2018). Volatile Fatty Acid Platform: Concept and Application. Emerging Areas in Bioengineering.

[48] Fei Q., Chang H.N., Shang L., dal rae Choi J., Kim N.J., Kang J.W. (2011). The effect of volatile fatty acids as a sole carbon source on lipid accumulation by Cryptococcus albidus for biodiesel production. Bioresour. Technol..

[49] Fradinho J.C., Oehmen A., Reis M.A.M. (2014). Photosynthetic mixed culture polyhydroxyalkanoate (PHA) production from individual and mixed volatile fatty acids (VFAs): Substrate preferences and co-substrate uptake. J. Biotechnol..

[50] Arous F., Azabou S., Triantaphyllidou I.E., Aggelis G., Jaouani A., Nasri M., Mechichi T. (2017). Newly isolated yeasts from Tunisian microhabitats: Lipid accumulation and fatty acid composition. Eng. Life Sci..

[51] Chaturvedi S., Bhattacharya A., Khare S.K. (2018). Trends in Oil Production from Oleaginous Yeast Using Biomass: Biotechnological Potential and Constraints. Appl. Biochem. Microbiol..

[52] Katre G., Joshi C., Khot M., Zinjarde S., Ravikumar A. (2012). Evaluation of single cell oil (SCO) from a tropical marine yeast yarrowia lipolytica NCIM 3589 as a potential feedstock for biodiesel. AMB Express.

[53] Chalima A., Hatzidaki A., Karnaouri A., Topakas E. (2019). Integration of a dark fermentation effluent in a microalgal-based biorefinery for the production of high-added value omega-3 fatty acids. Appl. Energy.

[54] Zhang S., Skerker J.M., Rutter C.D., Maurer M.J., Arkin A.P., Rao C.V. (2015). Engineering Rhodosporidium toruloides for Increased Lipid Production. Biotechnol. Bioeng..

[55] Qiao K., Wasylenko T., Zhou K., Xu P., Stephanopoulos G. (2017). Lipid production in Yarrowia lipolytica is maximized by engineering cytosolic redox metabolism. Nat. Biotechnol..

[56] Islam M.A., Yousuf A., Karim A., Pirozzi D., Khan M.R., Wahid Z.A. (2018). Bioremediation of palm oil mill effluent and lipid production by Lipomyces starkeyi: A combined approach. J. Clean. Prod..

[57] Viñarta S.C., Angelicola M.V., Barros J.M., Fernández P.M., Mac Cormak W., Aybar M.J., de Figueroa L.I.C. (2016). Oleaginous yeasts from Antarctica: Screening and preliminary approach on lipid accumulation. J. Basic Microbiol..

[58] Chatterjee S., Mohan S.V. (2018). Microbial lipid production by Cryptococcus curvatus from vegetable waste hydrolysate. Bioresour. Technol..

