# Supplementary material for: Single Cell Oil Production by Oleaginous Yeasts Grown in Synthetic and Waste-Derived Volatile Fatty Acids
Source: Microorganisms. 2020 Nov 17;8(11):1809. doi: 10.3390/microorganisms8111809 (PMC7698568; doi:10.3390/microorganisms8111809)
Supplement: Supplementary file 1 [file microorganisms-08-01809-s001.pdf]

Supplementary Material

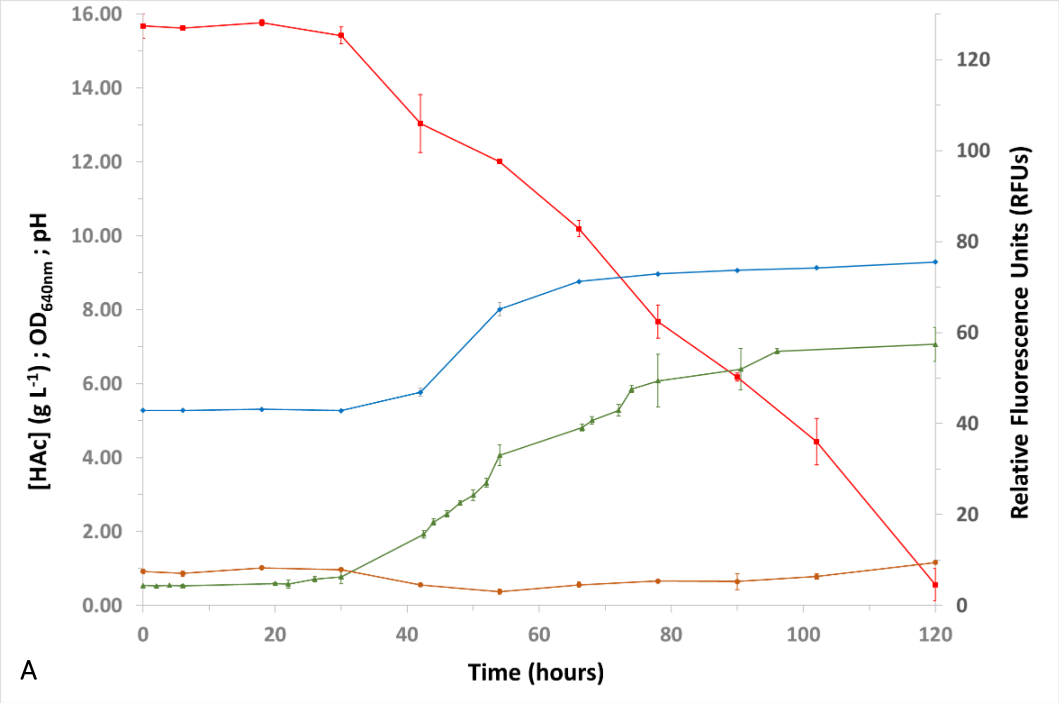

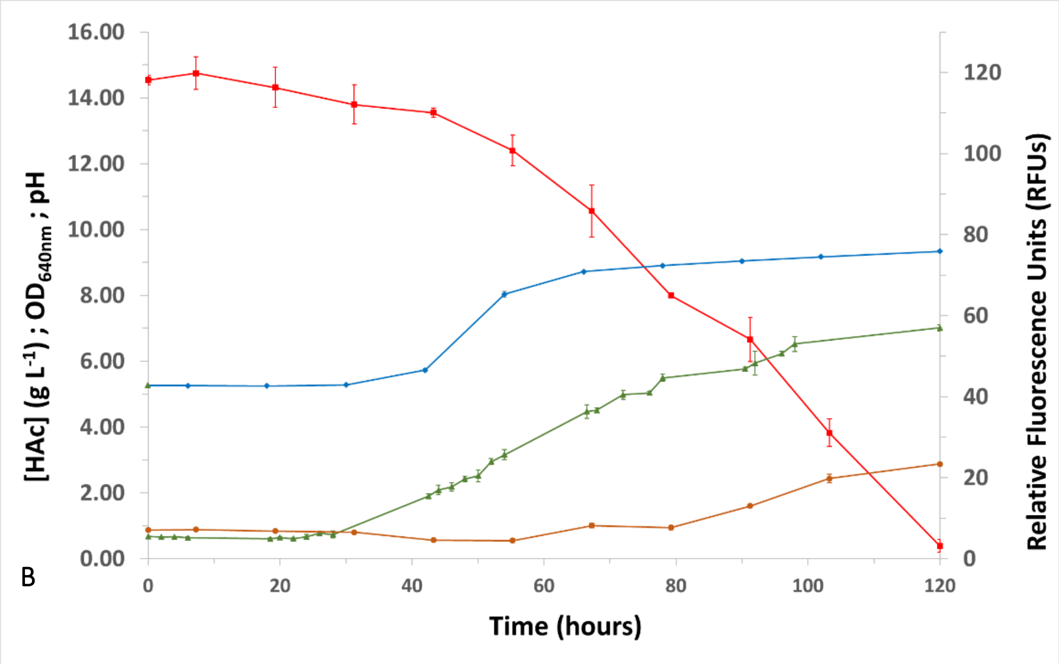

Figure S1: **Time-course growth in YP:HAc (15g L^{-1}) medium, during 120 hours, at pH 5.5**, using two of the four tested yeast species: **A**) *Candida tropicalis* V139; **B**) *Metchnikowia pulcherrima* V213.
Colours indicate measurements (mean ± standard deviations) of the following characteristics: Orange - relative fluorescent units (RFUs); Green – optical density (OD_{640}); Blue – pH; Red – acetic acid concentration (g L^{-1}).

Table S1: Biomass, lipid production and carbon source utilization in medium containing acetic acid (15 g L^{-1}) as carbon source, at pH 5.5, by two of the four selected yeast isolates in medium containing acetic acid (15 g L^{-1}) as carbon source, at pH 5.5.

$Y_{X/S}$ - growth yield (g DCW per g carbon added); $Y_{L/S}$ - lipid yield (g lipids per g carbon added).

Results represent mean of triplicates ± standard deviations.

	**DCW (g L^{-1})**	**Lipid content (%, w/w)**	**Lipid produced (g L^{-1})**	**$Y_{X/S}$ (g g^{-1} C_{added})**	**$Y_{L/S}$ (g g^{-1} C_{added})**	**Carbon source reduction (%)**
***C. tropicalis* V139**	3.36 ± 0.08	14.0 ± 0.3	0.47 ± 0.02	0.56 ± 0.01	0.078 ± 0.003	98
***M. pulcherrima* V213**	2.6 ± 0.2	21.0 ± 0.8	0.55 ± 0.05	0.43 ± 0.04	0.091 ± 0.008	97

Table S2: Long chain fatty acids profiles (%) obtained after 120 hours of growth in medium containing acetic acid (15 g L^{-1}), at pH 5.5, for two of the four selected yeast isolates.

Results represent mean of triplicates ± standard deviations.

	**C14:0**	**C15:0**	**C16:0**	**C16:1**	**C17:1**	**C18:0**	**C18:1n9**	**C18:2**	**C18:3**	**C20:0**	**Sum**
	**Myristic acid**	**Pentadecanoic acid**	**Palmitic acid**	**Palmitoleic acid**	**Heptadecenoic acid**	**Stearic acid**	**Oleic acid**	**Linoleic acid**	**γ-Linolenic acid**	**Eicosenoic acid**	
***C. tropicalis* V139**			13.5 ± 0.2	7.0 ± 0.1	1.2 ± 0.04	12 ± 0.5	57 ± 1	8.0 ± 0.3			98
***M. pulcherrima* V213**			13.3 ± 0.2	8.7 ± 0.2		9.5 ± 0.2	62.2 ± 0.1	4.1 ± 0.1			98

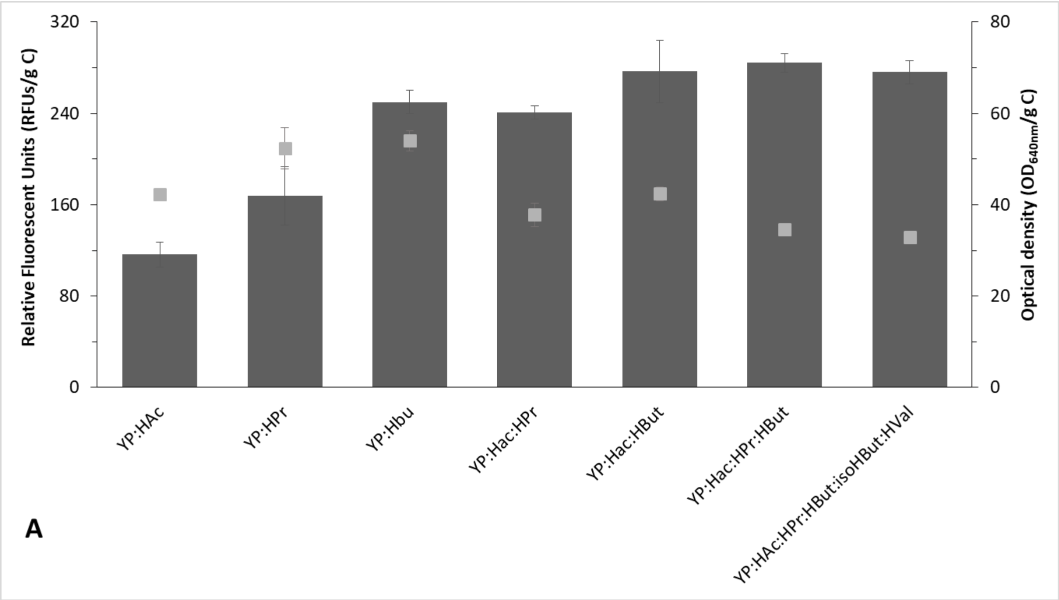
Relative Fluorescent Units (RFUs/g C)
Optical density (OD640nm/g C)
320
240
160
80
0
80
60
40
20
0
YP:HAc
YP:HPr
YP:Hbu
YP:Hac:HPr
YP:Hac:HBut
YP:Hac:HPr:HBut
YP:HAc:HPr:HBut:isoHBut:HVal
A

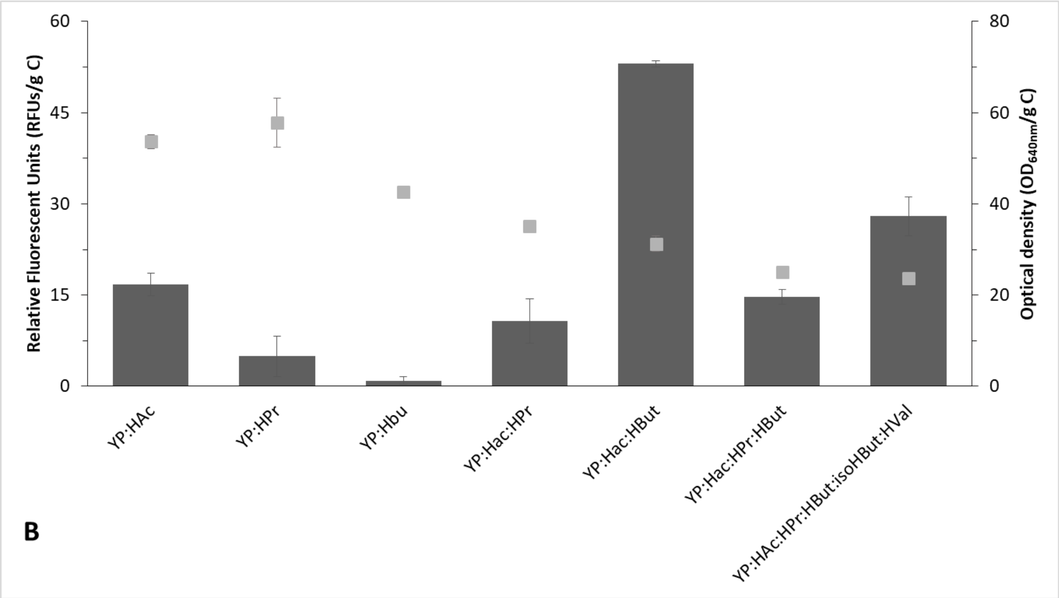
Relative Fluorescent Units (RFUs/g C)
Optical density (OD640nm/g C)
60
45
30
15
0
80
60
40
20
0
YP:HAc
YP:HPr
YP:Hbu
YP:Hac:HPr
YP:Hac:HBut
YP:Hac:HPr:HBut
YP:HAc:HPr:HBut:isoHBut:HVal
B

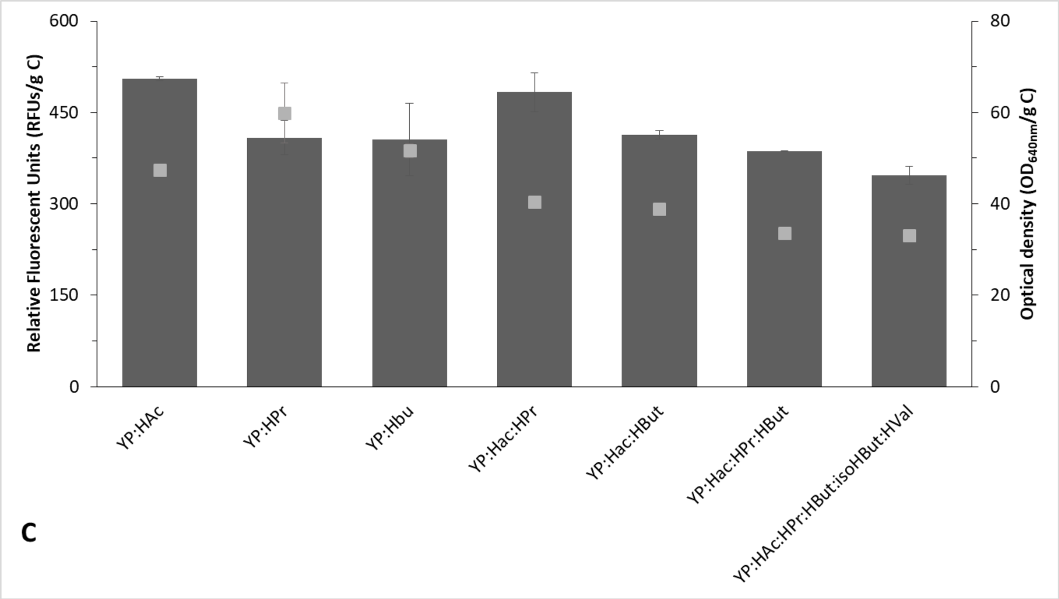

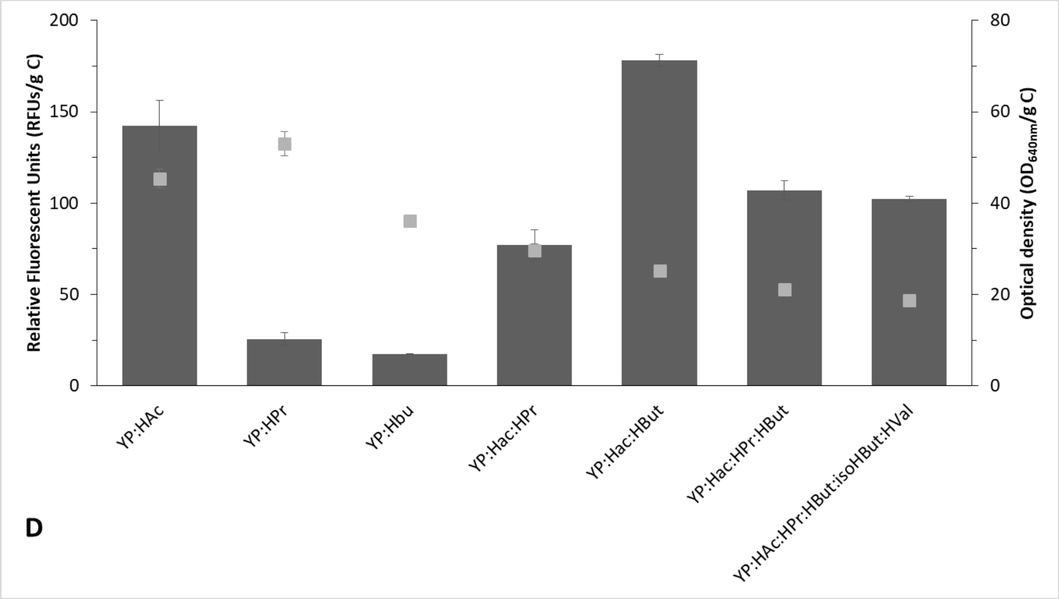

Figure S2: **Lipid production (relative fluorescent units, RFUs) and growth (OD_{640}) normalized per gram of carbon initially added in each medium, after 120 hours of growth** using the four selected yeast isolates: **A)** *Apiotrichum brassicae* V134; **B)** *Candida tropicalis* V139; **C)** *Metchnikowia pulcherrima* V213; **D)** *Pichia kudriavzevii* V194. Columns represent the amount of lipids accumulated and squares the optical density at 640 nm. The experiments were carried at least in duplicates.
